# Toward the Development of a Uranium‐Based Redox‐Flow Battery

**DOI:** 10.1002/cssc.202501782

**Published:** 2025-12-12

**Authors:** Pablo Waldschmidt, Nadir Jori, Judith Riedhammer, Frank W. Heinemann, Karsten Meyer

**Affiliations:** ^1^ Department of Chemistry and Pharmacy Inorganic Chemistry Friedrich‐Alexander‐Universität Erlangen‐Nürnberg (FAU) Erlangen Germany

**Keywords:** electrochemistry, f‐block, nonaqueous redox‐flow batteries (NARFBs), redox‐flow battery (RFB), uranium

## Abstract

An all‐uranium‐based electrochemical cell consisting of simple [U^IV/V^(^*t*Bu^acac)_4_]^0/+^ and [U^III/IV^(N(SiMe_3_)_2_)_4_]^−/0^ complexes as anolyte and catholyte species was constructed with a cell voltage of 2.2 V. The [U^IV^(^*t*Bu^acac)_4_] (**1**) and [U^IV^(N(SiMe_3_)_2_)_4_] (**2**) complexes have favorable properties for redox‐flow‐battery applications, including reversible redox chemistry, relatively high stability toward electrochemical cycling, and high solubility in common organic solvents. The [U^III/IV^(N(SiMe_3_)_2_)_4_]^−/0^ complexes were first isolated and characterized by Schelter et al., and performed well in electrochemical studies due to the comparably low reduction potential of −2.05 V vs. Fc/Fc^+^ to the reduced uranium(III) species. Treatment of conveniently accessible **1** with AgSbF_6_ allowed the isolation of [U^V^(^*t*Bu^acac)_4_][SbF_6_] (**3**), which is the active catholyte species generated during cell charging. Galvanostatic cycling with charging and discharging at currents of 20 and 5 μA, respectively, was performed in a two‐compartment static H‐cell with high‐surface‐area carbon fiber electrodes to achieve a potential of 2.2 V. The success of this **1**||**2** cell‐provides a promising entry point to a potential future class of uranium‐based, nonaqueous redox‐flow‐battery electrolytes, not for use in personal devices but incorporated into underground energy storage systems, where weight and radioactivity levels are not an issue and where this abundant waste material could find new application.

## Graphical abstract

The first all‐uranium‐based electrochemical cell has been realized using simple [U^IV/V^(^tBu^acac)_4_]^0/+^ and [U^III/IV^(N(SiMe_3_)_2_)_4_]^–/0^ complexes, readily available in convenient, scalable syntheses, as anolyte and catholyte couples. Operating at a cell potential of 2.2 V with a projected energy density of 0.357 Wh kg^–1^, this system establishes a foundation for the development of uranium‐based redox‐flow batteries.

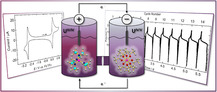

## 1 Introduction

Energy storage in the form of battery systems is playing an increasingly crucial role in the modern energy industry, where environmentally friendly, renewable energy sources are replacing reliable fossil fuels with unpredictable short‐term availability. Redox‐flow batteries (RFBs) have emerged as promising contenders due to their unique characteristics and versatile applications. They offer the ability to store and deliver electric energy efficiently while providing conveniently scalable, extended‐duration, and grid‐independent solutions [1, 2, 3, 4, 5, 6, 7, 8]. Aqueous vanadium‐ and zinc‐bromine‐based RFBs have successfully demonstrated reliable electrochemical performance at the commercial level [7, 9, 10, 11, 12, 13, 14].

Nonaqueous redox‐flow batteries (NARFBs) have emerged as a significant area of research due to several advantages, such as higher energy densities and greater flexibility in redox chemistry [15, 16, 17, 18]. Most importantly, NARFBs expand the operational voltage window from 1.2 V in water to windows up to >5 V, depending on the organic solvent [16, 17, 19, 20, 21, 22, 23, 24, 25, 26]. Nevertheless, NARFBs based on transition–metal complex electrolytes [13, 21, 27, 28, 29, 30, 31, 32, 33] have encountered various challenges, including limited cyclability [34], high production costs, high flammability, restricted electrochemical stability [24, 34], ligand dissociation [23, 24, 35], and cross‐contamination between the anolyte and catholyte compartments [22].

**FIGURE 1:**
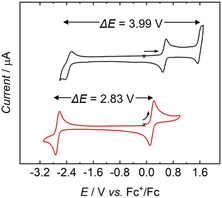
Cyclic voltammograms of [U^IV^((^*t*Bu,*t*Bu^ArO)_4_cyclen)] (black) and [U^IV^(^*t*Bu^acac)_4_] (**1**) (red) scanned at 200 mV/s and referenced vs. Fc/Fc^+^ (1 mM, MeCN). Working electrode, glassy carbon; pseudoreference electrode, Pt wire; counter electrode, Pt wire. The starting point of each CV cycle is marked with a cross, and the starting sweep direction with an arrow.

The drastic changes in coordination properties that most transition metal (d‐block) ions experience upon changes in oxidation state by more than one unit render it virtually impossible to stabilize one element within a stiff coordination environment in all oxidation states required for an RFB. Uranium, as an f‐block element, offers a unique solution to this challenge. Because the 5f orbitals are far less involved in bonding than the d orbitals of transition metals, the coordination geometry is correspondingly less rigid. This greater flexibility, combined with uranium's extraordinary redox and coordination chemistry, enables stabilization across the multiple oxidation states needed for RFB operation. Hence, molecular uranium species can efficiently stabilize the metal center in a wide range of oxidation states from +1 to +6 with minimal structural changes [36, 37, 38, 39, 40, 41, 42]. Combined with the large potential ranges of readily accessible redox couples, such as U^II/III^, U^III/IV^, U^IV/V^, and U^V/VI^, unusually high cell potentials can be achieved in organic solvents (Figure 1) [43, 44, 45]. Moreover, there is a pressing need for the use of depleted uranium from nuclear reactors, 95% of which is mostly stored in the form of highly corrosive UF_6_ in relatively unprotected bins [46].

Here, we report a novel approach to NARFB electrolyte design using redox‐active uranium complexes. The presented system is based on the previously reported U(IV) complexes, [U^IV^(^*t*Bu^acac)_4_] (**1**) and [U^IV^(N(SiMe_3_)_2_)_4_] (**2**), where ^*t*Bu^acac^–^ = 2,2,6,6‐tetra‐methyl‐3,5‐heptanedionate, and N(SiMe_3_)_2_^–^ = *bis‐*(trimethylsilyl)‐amido. Both complexes are known for their stability and electrochemical reversibility. Complex **2**, in particular, has gained significant recognition as a prominent precursor in uranium chemistry since its publication by Schelter et al*.* in 2013 [47]. Pairing **1** and **2** as anolyte and catholyte species results in a nominal cell voltage of 2.2 V. The metal complexes are neutral in the discharged state of the RFB, minimizing crossover through the membrane separator. Additionally, the high solubility of these uranium complexes in organic media offers a very interesting entry point for developing NARFBs. The notable solubility coupled with low cost, convenient accessibility, and increased stability render **1** and **2** promising building blocks for RFB chemistry [21, 22, 25, 27, 45, 47].

## 2 Results and Discussion

Our previous electrochemical studies on the redox complex pairs [U((^*t*Bu,*t*Bu^ArO)_4_cyclen)] [43] and [U(^*t*Bu^acac)_4_] [45], which exhibited electrochemical windows of up to ca. 4 V, sparked the idea of designing an RFB based on a single compound capable of undergoing simultaneous oxidation at the anode and reduction at the cathode. This concept, envisioned as an entry point toward a long‐term goal, aimed to address a major lifetime‐limiting factor, namely diffusion. However, the exceedingly large reduction potentials, approaching the limit of solvent stability, hindered its immediate implementation. We therefore turned to a dual‐active‐species electrochemical cell.

The homoleptic acac complex, [U^IV^(^*t*Bu^acac)_4_] (**1**), was chosen for this project, following the established use of various transition metal complexes using this ligand framework [21, 22, 48, 49, 50]. Although earlier reports indicate the instability of the corresponding uranium(III) species [45], complex **1** remains an excellent candidate for an anolyte material, thus taking advantage of its U^IV/V^ redox couple due to its high solubility, remarkable stability in solution, and convenient availability. Consequently, the primary challenge toward a practical battery application lies in identifying a uranium complex with a suitable electrochemical potential for the U^III/IV^ couple. Most reductions to trivalent uranium occur at potentials well below –2.5 V vs. Fc/Fc^+^, which poses a problem as such reduced molecules often show significant instability in solution, particularly over extended periods of time [44]. Among numerous tetravalent uranium complexes, [U^IV^(N(SiMe_3_)_2_)_4_] (**2**) stands out as an exceptional catholyte material with favorable accessibility and an electrochemical reduction potential of –2.05 V vs. Fc/Fc^+^.

Treatment of **1** with AgSbF_6_ allowed for the isolation of the one‐electron‐oxidized complex, [U^V^(^*t*Bu^acac)_4_][SbF_6_] (**3**), which is the active catholyte species generated during this cell charging. [U^IV^(^*t*Bu^acac)_4_] and [U^V^(^*t*Bu^acac)_4_][SbF_6_] were synthesized and isolated as crystalline solids. The latter was characterized by a combination of UV/vis/NIR (Figures S3 and S4), ^1^H NMR (Figure S2), EPR spectroscopy (Figure S13), sc‐XRD (Figure S23), and SQUID magnetization studies (Figure S12) [49]. [U^IV^(N(SiMe_3_)_2_)_4_] (**2**) was synthesized according to the literature. Tetravalent **4** can be reduced in various ways, for example, utilizing potassium as a reducing agent to isolate [K(THF)_6_][U^III^[N(SiMe_3_)_2_]_4_] (**4**), which resembles the active anolyte species [47, 51, 52].

As a polar organic solvent, acetonitrile (MeCN) proved to be ideal for the electrolytes [U^IV^(^*t*Bu^acac)_4_] (**1**) and [U^IV^(N(SiMe_3_)_2_)_4_] (**2**), as it is commonly used for NARFB applications based on its wide electrochemical window (∼5 V) and low viscosity [53]. The solubility of **1** and **2** in MeCN was determined to be 0.567 M and 0.093 M, respectively (Figures S5–S10), resulting in a potentially achievable energy density of 0.0375 Wh/kg. This solubility is about six times lower than the classical vanadium RFB's solubility of 3.3 M in water [48] but is comparable to or better than other transition metal complex electrolytes [23]. Additionally, it surpasses the operational concentration of V(acac)_3_ (around 50 mM) by a factor of ∼2–10 [54].

**FIGURE 2:**
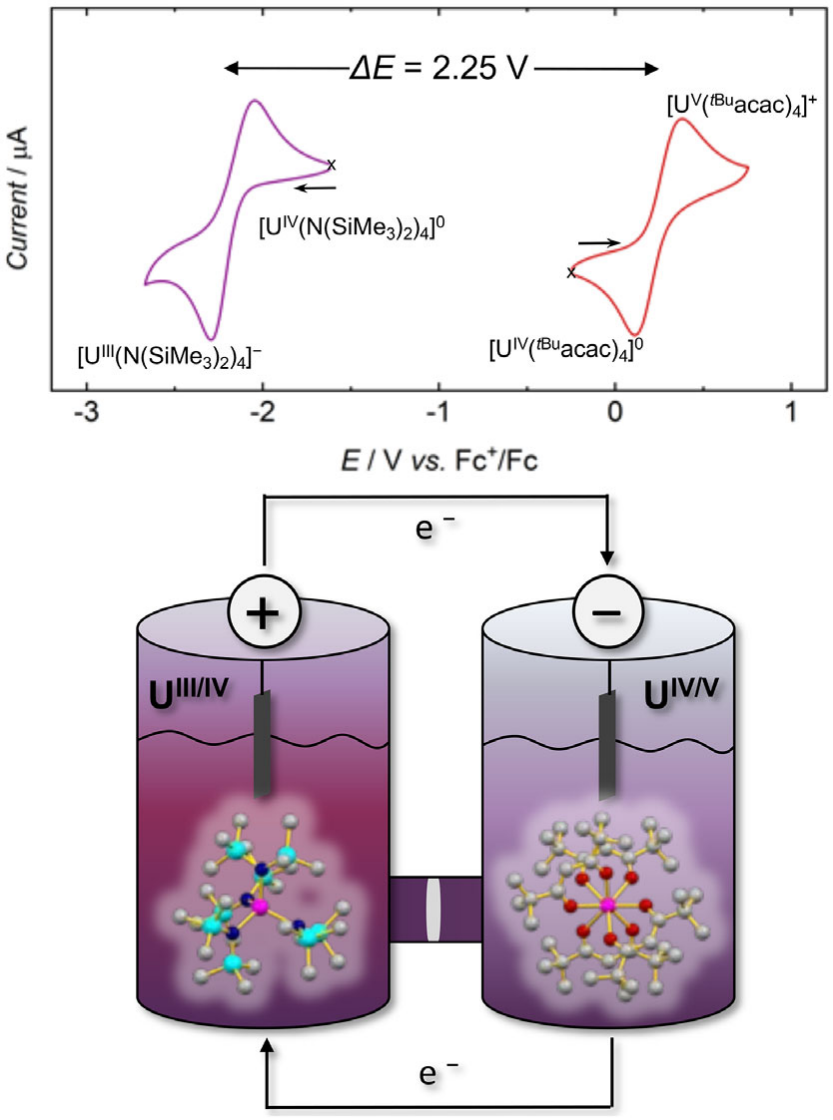
(Top) Cyclic voltammograms of [U^IV^(N(SiMe_3_)_2_)_4_ (**2**) (purple) and [U^IV^(^*t*Bu^acac)_4_] (**1**) (red), scanned at 200 mV/s and referenced vs. Fc/Fc^+^ (1 mM, MeCN). Working electrode, glassy carbon; pseudoreference electrode, Pt wire; counter electrode, Pt wire. The starting point of each CV cycle is marked with a cross, and the starting sweep direction with an arrow. (Bottom) Schematic representation of an H‐cell used for charge−discharge studies, with molecular structure representations of [U^IV^(N(SiMe_3_)_2_)_4_]^−/0^ (catholyte, left) and [U^IV^(^*t*Bu^acac)_4_]^0/+^ (anolyte, right). Typical experiments were run at 1 mM [U^IV^(N(SiMe_3_)_2_)_4_] and [U^IV^(^*t*Bu^acac)_4_] concentrations in a 0.5 M MeCN solution of [N(^n^Bu)_4_][PF_6_], with a Neosepta ACS anion‐selective membrane separating the compartments and carbon electrodes.

In acetonitrile, tetravalent **1** undergoes a reversible oxidation at *E*_1/2_ = +0.16 V vs. Fc/Fc^+^, which is attributed to the U^IV/V^ couple. In contrast, tetravalent **2** displays a reversible reduction at *E*_1/2_ = −2.05 V vs. Fc/Fc^+^, which is assigned to the U^IV/III^ couple (Figure 2, top).

The combination of these reversible redox events enables the construction of a cell with a theoretical voltage of 2.2 V. In this configuration, **1** works as the anolyte, cycling between U(IV) and U(V), while **2** serves as the catholyte, cycling between U(IV) and U(III). Within this system, both metal complexes undergo one‐electron redox events, alternating between neutral and mono‐ionic species (Figure 2, bottom).

**FIGURE 3:**
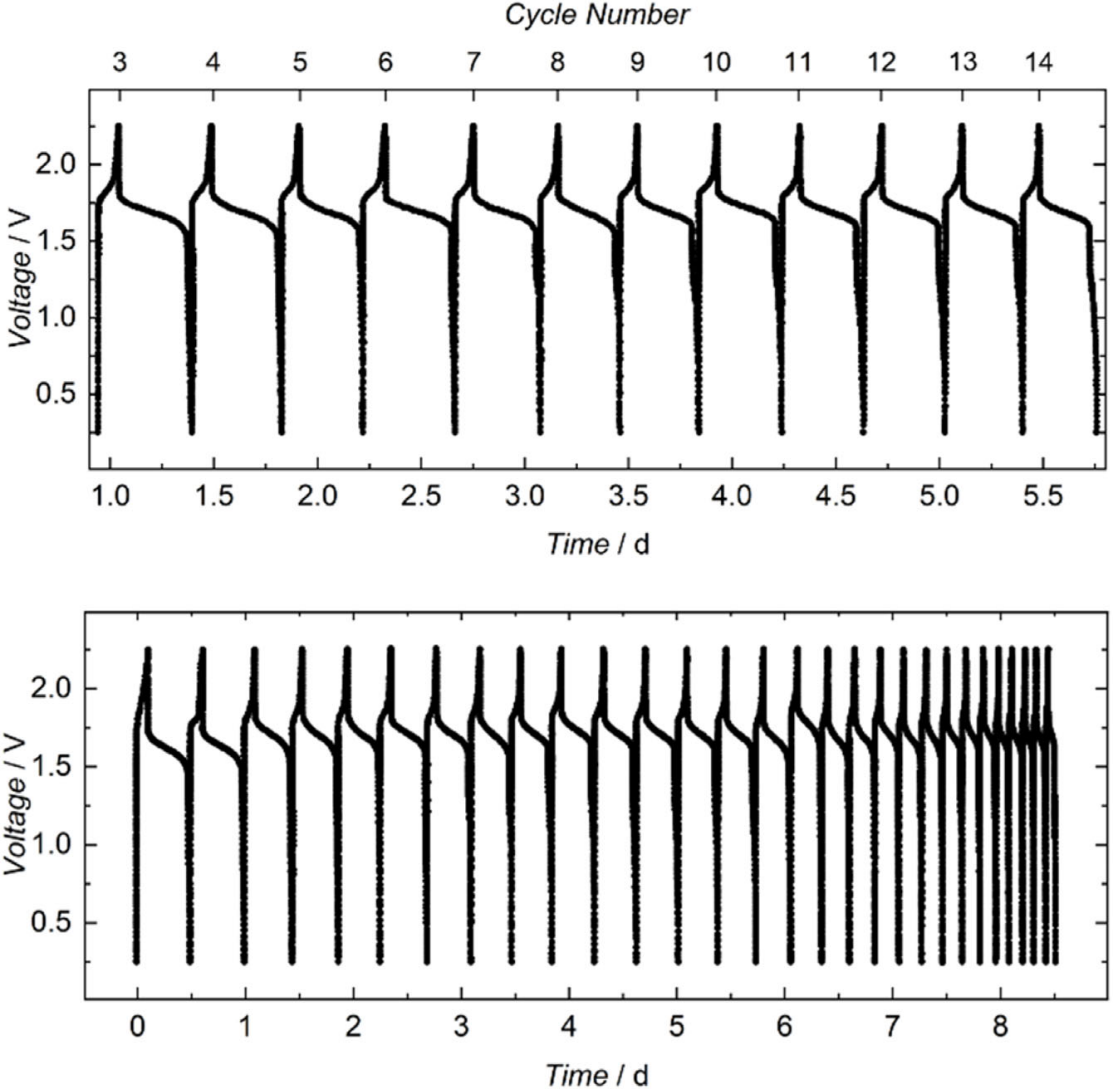
Top: Representative galvanostatic voltage vs. time profile recorded between the 3rd and 14th cycle of the [U^IV^(N(SiMe_3_)_2_)_4_](+) || [U^IV^(^*t*Bu^acac)_4_](−) cell (1 mM in MeCN) at a charge rate of 20 μA and a discharge rate of 5 μA. The charging and discharging currents were measured by applying a constant voltage to the cell and recording the system's current during charge (20 μA) and discharge (5 μA). Bottom: Representative voltage vs. time profile recorded between the 1st and 29th cycle of the [U^IV^(N(SiMe_3_)_2_)_4_](+) || [U^IV^(^*t*Bu^acac)_4_](−) cell (1 mM in MeCN).

A two‐compartment static H‐cell was constructed to evaluate the charge–discharge characteristics of **1** and **2** in MeCN. This H‐cell, commonly used to simulate flow system conditions, allows for the use of small volumes of electrolyte solutions [23]. The two compartments were separated by a Neosepta ACS (ASTOM, Japan) membrane (Figure 2, bottom), an anion‐exchange membrane with mono‐anion selectivity. This membrane is expected to allow the transport of [PF_6_]^−^ anions [55, 56], while minimizing permeation of the redox‐active species, [U^IV/V^(^*t*Bu^acac)_4_]^0/+^ and [U^III/IV^(N(SiMe_3_)_2_)_4_]^−/0^. Electrodes of high‐surface‐area carbon fiber (with 1 cm^2^ macroscopic area) were used in each compartment. Galvanostatic cycling of the cell was performed with charging and discharging at currents of 20 and 5 μA, respectively (Figure 3, top). Voltage cutoffs (0.25–2.2 V) were determined based on cyclic voltammograms (CVs) of each complex to ensure selective access to the desired redox couples. Notably, the recorded operational voltage of 2.2 V (Figure 2, top) ranks within the highest category of the latest advancements in NARFB technology [13, 21, 23, 27, 31, 34, 49, 57, 58, 59, 60, 61, 62, 63].

**FIGURE 4:**
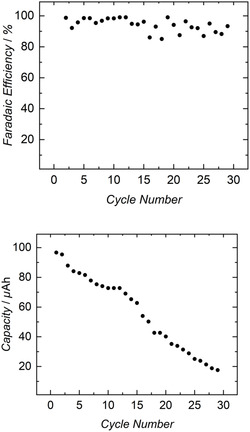
Top: Faradaic Efficiency vs. cycle number for all 29 cycles of the **1**||**2** cell. Bottom: Capacity vs. cycle number for all 29 cycles of the **1**||**2** cell.

The **1**||**2** cell exhibited charge–discharge cycles for up to 29 cycles (Figure 3, bottom), with notably diminishing capacity after 7 days. Hence, we derived capacity values from the galvanostatic curves to examine the limitations of the **1**||**2** cell. Analysis of capacity plotted against the cycle number (Figure 4, bottom) revealed a nearly linear decrease over time. The averaged single‐cycle Faradaic efficiency was calculated to be 94%, corresponding to 15% capacity retention over 29 cycles. This consistent decrease in capacity required further investigation. Subsequently, the anolyte and catholyte materials were tested after 29 charge–discharge cycles to reveal minor decomposition of the complexes in both cells.

This decomposition was indicated by the presence of free ligand signals in the ^1^H NMR spectra of both compartments, as well as the signal of [U^IV^(N(SiMe_3_)_2_)_4_] in the anolyte solution, confirming catholyte diffusion through the membrane (Figures S17–S18). Moreover, additional investigations were conducted on the graphite electrodes utilized in the H‐cell setup (Figures S19–S20). Cyclic voltammetry analysis of the electrodes before and after 29 charge–discharge cycles revealed a significantly broadened CV, typical for deposition on the electrode surface. The relatively low current density is a limiting factor, as the experiments employed low concentrations to ensure the production of high‐quality datasets. We note, however, that increasing the concentration of the electrolyte solutions could still enhance the energy density (up to approximately 3.451 Wh kg^–1^, see Supporting information). By contrast, another limitation arises from the sluggish kinetics of the oxidation of [U^III^(N(SiMe_3_)_2_)_4_]^–^ back to [U^IV^(N(SiMe_3_)_2_)_4_], as evident from the CV of this compound, where the oxidation wave of the U(IV)/U(III) redox couple shifts to higher potentials at a scan rate of 200 mV s^–1^. The decomposition of cell components was tracked and identified through the analysis of the NMR spectra (Figures S17 and S18).

Also, while the present study provides a proof‐of‐concept, we recognize that any large‐scale application of uranium‐based electrolytes must carefully evaluate environmental and sustainability aspects. Although depleted uranium is primarily an *α*‐emitter, it poses a risk of groundwater contamination if released. Consequently, robust engineering solutions—durable encapsulation, corrosion‐resistant casings, and long‐term monitoring—would be essential, and these requirements must be assessed within a comprehensive life‐cycle sustainability framework. At the same time, uranium's unique redox flexibility presents an exciting opportunity: U‐based NARFB systems could, in principle, achieve operating potentials of up to 4 V [43], far surpassing those of many existing flow‐battery chemistries. With appropriate technological development, such systems could turn an abundant nuclear waste material into the foundation of a powerful, sustainable energy storage technology.

## 3 Conclusion

The electrochemical reduction of water to hydrogen gas using a *tris*‐aryloxide uranium(III) system was a paradigm shift, representing the first electrocatalytic process for an element in the actinide series [64, 65]. This can be considered the first application of uranium complexes to potentially sustainable energy conversion applications, the feasibility of which had been doubted in the past. The next milestone for uranium chemistry would be the integration of its molecular compounds into the fundamental components of RFBs. Depleted uranium‐based RFBs could be incorporated into underground energy storage systems, e.g., within the heavy concrete foundations of wind turbines, where weight and radioactivity levels are not an issue and where this abundant waste material from the nuclear industry could find a new application. Accordingly, this study introduces the first example of an all‐uranium‐based electrochemical cell, utilizing the conveniently accessible and large‐scale synthesizable [U^IV/V^(^*t*Bu^acac)_4_]^0/+^ and [U^III/IV^(N(SiMe_3_)_2_)_4_]^−/0^ complexes as anolyte and catholyte species, respectively. The constructed cell achieved a notable voltage of 2.2 V and an energy density of 0.0375 Wh/kg. The simple and stable tetravalent [U^IV^(^*t*Bu^acac)_4_] (**1**) and [U^IV^(N(SiMe_3_)_2_)_4_] (**2**) complexes show favorable properties for RFB applications, including reversible redox chemistry with high reversibility toward electrochemical cycling and high solubility in common organic solvents. The success of the **1**||**2** cell marks a significant milestone, offering a promising future class of uranium‐based, NARFB electrolytes.

In summary, the **1**||**2** cell demonstrated charge–discharge cycles for up to 29 cycles. Over these cycles, it was observed that the catholyte, most likely in its reduced, mono‐anionic state, diffuses through the membrane. Additionally, minor decomposition of both complexes occurred during the experiment, and deposition on the electrodes was observed. We have addressed the stability of the anolyte and catholyte species within the H‐cell configuration and performed capacity studies. However, further investigations are required to establish stability benchmarks relevant to an operational flow cell. While the present system exhibits apparent limitations, it nonetheless represents an exciting entry point toward the development of improved all‐uranium RFBs and warrants further investigation and optimization.

Despite these challenges, the results still support the use of the **1**||**2** system in RFB applications. The membrane remains the weak link in the current setup, motivating future efforts toward either improved separators or membrane‐free architectures, e.g., based on orthogonal solubilities. Future efforts will focus on conducting more comprehensive studies on cell stability and efficiency, as well as optimizing conditions for an operational single‐compound, membrane‐less flow cell. We acknowledge that our departure from the initial design of an RFB, which consisted of only a single compound to address diffusion issues, diminishes the original approach. However, it remains plausible that our core findings reported here could be adapted to other uranium‐based complexes, rendering the results presented here both novel and significant.

Thus, this study introduces the first all‐uranium‐based RFB as a proof‐of‐concept, highlighting both the promise and the challenges of such systems. While large‐scale applications will require careful consideration of sustainability and engineering aspects, the exceptional redox flexibility of uranium offers the prospect of nonaqueous flow batteries with unusually large operating potentials, marking a significant advance for future energy‐storage technologies.

## Supporting Information

Additional supporting information can be found online in the Supporting Information section. **Supporting Fig. S1:** Representative picture of an Fe(III) sample, loaded within a polycarbonate gel capsule inside a plastic straw. **Supporting Fig. S2:** ^1^H NMR spectrum of [U^V^(^tBu^acac)_4_][SbF_6_] (**3**) in THF‐*d*_8_ (#). **Supporting Fig. S3:** Vis/NIR electronic absorption spectrum of [U^V^(^*t*Bu^acac)_4_][SbF_6_] (**3**) in THF, c = 10 mM. **Supporting Fig. S4:** UV/Vis electronic absorption spectrum of [U^V^(^*t*Bu^acac)_4_][SbF_6_] (**3**) in THF, c = 0.1 mM. **Supporting Fig. S5:** UV–vis spectra of [U^IV^(^tBu^acac)_4_] (**1**) in MeCN at concentrations in the range 10‐50 µM. **Supporting Fig. S6** **:** Calibration curve of absorbance as a function of concentration of [U^IV^(tBuacac)_4_] (**1**) in MeCN. **Supporting Fig. S7:** UV–vis spectra of five independent samples of [U^IV^(^tBu^acac)_4_] (**1**), prepared by dilution (1:24,000) of a saturated solution in MeCN. **Supporting Fig. S8:** UV–vis spectra of [U^IV^(N(SiMe_3_)_2_)_4_] (**2**) in MeCN at concentrations in the range 50‐150 µM. **Supporting Fig. S9:** Calibration curve of absorption as a function of concentration of [U^IV^(N(SiMe_3_)_2_)_4_] (**2**) measured in MeCN. **Supporting Fig. S10:** UV–vis spectra of 5 independent samples of [U^IV^(N(SiMe_3_)_2_)_4_] (**2**) prepared by dilution (1:600) of a saturated solution in MeCN. **Supporting Fig. S11:** Temperature‐dependent SQUID magnetization measurements of [U^IV^(^tBu^acac)_4_] (**1**); two independently synthesized and studied samples. Data reproduced from ref. [1]. **Supporting Fig. S12:** Temperature‐dependent SQUID magnetization measurements of [U^V^(^tBu^acac)_4_][SbF_6_] (**3**); two independently synthesized and studied samples. **Supporting Fig. S13:** CW X‐Band EPR spectrum of [U^V^(^tBu^acac)_4_][SbF_6_] (**3**), recorded at 6 K as a powder (ν = 8.96776 GHz, P = 1.0 mW, modulation = 0.1 mT). The spectrum was simulated with g values of g_1_ = 3.10 and line widths of W_1_ = 12 mT. The g_2_ and g_3_ values lie outside the experimentally accessible range and were set to arbitrary values for simulation. **Supporting Fig. S14:** Photographs of the H‐cell setup. Initial cell (left), and cell during charging (right). **Supporting Fig. S15:** Charge–discharge profile of the 1^st^–29^th^ cycle of the [U^IV^(N(SiMe_3_)_2_)_4_](+) | [U^IV^(^tBu^acac)_4_](−) (**1**||**2**) cell. Galvanostatic cycling of the cell was performed at 20 µA charge/ 5 µA discharge rate at 25°C under an atmosphere of purified N_2_. Conditions: anolyte, 0.02 mM [U^IV^(^tBu^acac)_4_] in a 0.1 M MeCN solution (5 mL) of [TBA][PF_6_]; catholyte, 0.02 mM [U^IV^(N(SiMe_3_)_2_)_4_] in a 0.1 M MeCN solution (5 mL) of [TBA][PF_6_]. A NeoseptaACS® (ASTOM, Japan) anion‐exchange membrane was used to separate each compartment of the H‐cell, and each side was equipped with a carbon paper electrode (2 cm^2^ surface area). **Supporting Fig. S16:** Charge–discharge profile during the 3^rd^ to 14^th^ cycles of the **1**||**2** cell (5 mM in MeCN). **Supporting Fig. S17:** ^1^H NMR spectra of the anolyte solution in benzene‐d_6_ (#) before (green) and after (red) 29 charge–discharge cycles. **Supporting Fig. S18:** ^1^H NMR spectra of catholyte solution in benzene‐d_6_ (#) before (green) and after (red) 30 charge–discharge cycles. **Supporting Fig. S19:** Cyclic voltammogram of Ferrocene with the carbon electrode used in the **1**||**2** cell before 30 charge–discharge cycles, measured in a MeCN solution with ∼0.1 M [N(^n^Bu)_4_][PF_6_] as an electrolyte; scan rate = 200 mV/s. **Supporting Fig. S20:** Cyclic voltammogram of ferrocene with the carbon electrode used in the **1**||**2** cell after 30 charge–discharge cycles measured in a MeCN solution with ∼0.1 M [N(^n^Bu)_4_][PF_6_] as an electrolyte; scan rate = 200 mV/s. **Supporting Fig. S21:** Cyclic voltammograms of [U^IV^(^tBu^acac)_4_] measured in a benzene/MeCN (1:1) solution with ∼0.1 M [N(^n^Bu)_4_][PF_6_] as an electrolyte, including the 2nd scan (black) and the 100th scan (red); scan rate = 200 mV/s. Data reproduced from ref. [1]. **Supporting Fig. S22:** Cyclic voltammograms of [U^IV^(N(SiMe_3_)_2_)_4_] measured in a MeCN solution with ∼0.1 M [N(^n^Bu)_4_][PF_6_] as an electrolyte, including the 2nd scan (black) and the 100th scan (red); scan rate = 200 mV/s. **Supporting Fig. S23:** Solid‐state molecular structure with the applied numbering scheme of **3** in crystals of [U^V^(^tBu^acac)_4_][SbF_6_] (top left: major component of the [U^V^(^tBu^acac)_4_] cation, top left: minor component of the [U^V^(^tBu^acac)_4_] cation, bottom: the SbF_6_ anion with the minor component of the disordered atoms drawn with dashes. Thermal ellipsoids are at the 50% probability level, hydrogen atoms are omitted for clarity). **Supporting Table S1:** Selected Interatomic Distances (Å) and Angles (deg) of [U^V^(^t^ ^Bu^acac)_4_][SbF_6_] (**3**). **Supporting Table S2:** Crystallographic data, data collection, and refinement details for **3**.

## Conflicts of Interest

The authors declare no conflicts of interest.

## Supporting information

Supplementary Material

## Data Availability

The data that support the findings of this study are available from the corresponding author upon reasonable request.
