# Supplementary material for: Toward the Development of a Uranium‐Based Redox‐Flow Battery
Source: ChemSusChem. 2025 Dec 12;19(1):e202501782. doi: 10.1002/cssc.202501782 (PMC12767275; doi:10.1002/cssc.202501782)
Supplement: Supplementary file 1 — Supplementary Material [file CSSC-19-e202501782-s001.pdf]

Supporting Information for the Paper Entitled:

Towards the Development of a Uranium-Based Redox-Flow Battery

Pablo Waldschmidt, Nadir Jori, Judith Riedhammer, Frank W. Heinemann and Karsten Meyer*

Friedrich-Alexander-Universität Erlangen-Nürnberg (FAU), Department of Chemistry and Pharmacy, Inorganic Chemistry, Egerlandstraße 1, 91058 Erlangen, Germany

Table of Contents

General Considerations S3

Synthetic and Spectroscopic Details S6

NMR Spectroscopy S7

UV/Vis/NIR Electronic Absorption Spectroscopy S8

Solubility Determination of $[U^{IV}(^{tBu}acac)_4]$ (1) and $[U^{IV}\{N(SiMe_3)_2\}_4]$ (2) S9

VT SQUID Magnetization Data S15

EPR Spectroscopic Measurements S16

Electrochemical Details S17

General procedure for bulk electrolysis studies. S17

Investigations into the cause of cell capacity decay S20

X-Ray Crystal Structure Determination S25

References S29

General Considerations

All air- and moisture-sensitive experiments were performed under dry nitrogen atmosphere, using standard Schlenk techniques or an MBraun inert-gas glovebox, containing an atmosphere of purified dinitrogen. The glovebox was equipped with a – 35 °C freezer.

Solvents were purified using a two-column solid-state purification system (Glass Contour System, Irvine, CA), transferred to the glovebox without exposure to air and moisture, and stored over activated molecular sieves and potassium, where appropriate (*n*-pentane, *n*-hexane, benzene). Commercially available starting materials were purchased from prominent suppliers (Acros Organics, Alfa Aesar, Sigma Aldrich, Merck, TCI, VWR) and were used without further purification. NMR solvents were obtained packaged under argon and stored over activated molecular sieves.

The complexes $[U^{IV}(^{tBu}acac)_4]$,[1] and $[U^{IV}\{N(SiMe_3)_2\}_4]$ [2] were prepared according to literature procedures.

NMR spectra were recorded on JEOL ECX 270 MHz or JEOL ECX 400MHz instruments, operating at respective frequencies of 269.71 MHz and 400.18 MHz (^{1}H NMR with a probe temperature of 23 °C. Chemical shifts, δ, are reported relative to the ^{1}H resonances of the solvent in ppm. [3]

NMR multiplicities are abbreviated as follows: s = singlet, m = multiplet, br = broad signal.

Electronic absorption spectra were recorded from λ = 200 nm to λ = 2200 nm on a Shimadzu double beam UV-3600 UV/Vis/NIR spectrophotometer in the given solvents at room temperature. Cuvettes of the type 117.100-QS from Hellma with 10 mm layer thickness were used. Data points between 1700 and 1750 nm are obscured due to the switch from the In/Ga/As detector to the PbS detector of the instrument and were removed from the graphical representation.

Magnetism data of powdered samples (10.0–25.0 mg), loaded within a polycarbonate gel capsule inside a plastic straw (**Figure S1**) were collected on a Quantum Design

MPMS-3 SQUID magnetometer. The DC moment was recorded in the temperature range of 2–300 K with an applied DC field of 1 T, if not stated otherwise. The DC moment was converted into molar magnetic susceptibility (χ_M) using the following formula (with H = magnetic field, n = moles of substance):

$$\chi_M = \frac{DC\ moment}{H \cdot n}$$

Values of the magnetic susceptibility were corrected for core diamagnetism (μ_{eff}) of the sample and Eicosane® (if used), using tabulated Pascal's constants.[4] Effective magnetic moments (μ_{eff}) were calculated using the following formula (with temperature (T)):

$$\mu_{eff} = 2.828 \cdot \sqrt{(\chi_M - \chi_{dia}) \cdot T}$$

For data simulation and analysis, the program "JulX2", written by Dr. Eckhard Bill (MPI CEC, Mülheim/Ruhr) was used.[5]

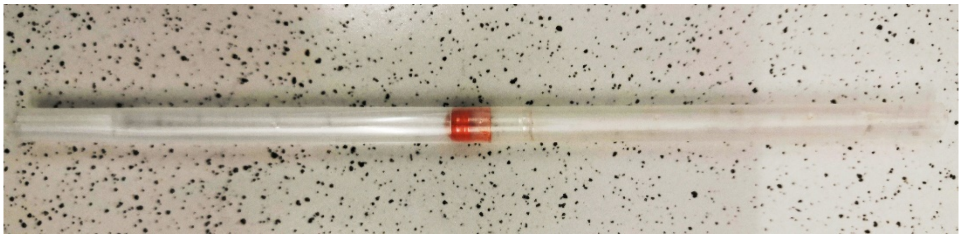

Figure S1: Representative picture of an Fe(III) sample, loaded within a polycarbonate gel capsule inside a plastic straw.

Magnetic susceptibility data in solution were measured by the Evans NMR method.[6] The outer tube (standard NMR tube with a PTFE valve) contained the reference solvent mixture THF/THF-d_8/TMS (10:2:1). The inner tube (capillary tube with 1.5 mm diameter sealed with inert wax) contained a $5 \cdot 10^{-4}$ M solution of the paramagnetic complex in the same solvent mixture.

EPR spectra were recorded on a JEOL continuous-wave spectrometer JES-FA200, equipped with an X-band Gunn diode oscillator bridge, a cylindric mode cavity, and a helium cryostat. If not stated otherwise, the samples were measured in the solid-state under nitrogen atmosphere in quartz glass EPR tubes at 293, 95, and 7 K. The spectra

shown were measured using the following parameters: microwave frequency = 8.959 GHz, modulation width 1.0 mT, microwave power 1.0 mW, modulation frequency 100 kHz, time constant of 0.1 s. Data analysis and simulation was performed using the software “eview” and “esim”, written by Dr. Eckhard Bill (MPI CEC, Mülheim/Ruhr),[7,8] on the basis of a spin-Hamiltonian description of the electronic ground state:

$$\widehat{H} = D\left(\hat{S}_z^2 - \frac{1}{3}S(S+1) + \frac{E}{D}\left(\hat{S}_x^2 - \hat{S}_y^2\right)\right) + \mu_B \underline{g}\vec{S}\ .$$

Here, S represents the total spin quantum number of the coupled system, D and E/D are the axial and rhombic zero-field parameters, respectively, and $\underline{g}$ is the g-matrix. Calculations are based on the S = 5/2 routines developed by Gaffney and Silverstone.[9] EPR line widths, W, are given in units of mT at full-width-half-maximum (FWHM).

Electrochemical measurements were carried out at room temperature under dinitrogen atmosphere with an μAutolab Type III potentiostat. Samples were recorded in 1 mM solutions of the analyte in acetonitrile, containing 0.1 M n-Bu_4NPF_6 (TBAPF6, ≥99.0% for electrochemical analysis, purchased from Sigma Aldrich and used without further purification) using a rotating disk electrode (for cyclovoltammetry) with a glassy carbon, platinum or gold tip (3 mm diameter) as working electrode and platinum wires as counter and pseudo-reference electrodes. Ferrocene (Fc) was added as an internal standard, and all measurements were referenced to the Fc^+/Fc couple.

Caution! Natural uranium is a radioactive α-emitter; and thus, direct and prolonged exposure and inhalation must be prevented. Manipulations should be carried out with care in monitored fume hoods or in inert atmosphere gloveboxes in radiation-controlled laboratories equipped with appropriate radiation counting instruments.

Synthetic and Spectroscopic Details

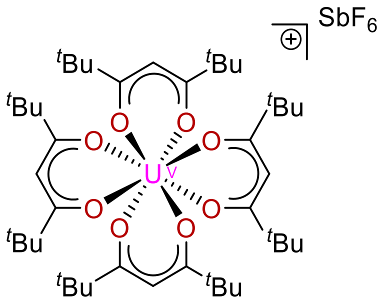

Synthesis of [U^{V}(^{tBu}acac)$_4$][SbF_6] (3): A solution of [U^{IV}(^{tBu}acac)$_4$] (**1**) (50 mg, 0.0508 mmol, 1 equiv.) in 3 mL of benzene was added to silver hexafluoroantimonate (17,5 mg, 0.0508 mmol, 1 equiv.). The reaction mixture was stirred for 1 hour and turned brown. The mixture was filtered, and the solvent was removed under vacuum. The remaining solid was washed three times with 3 mL *of n*-hexane to yield the product as a brown powder (30 mg, 0.025 mmol, 49% yield).

^{1}H NMR (400 MHz, THF-d_8): δ = 0.68 (s), –2.01 (s) ppm (Figure S2).

NMR Spectroscopy

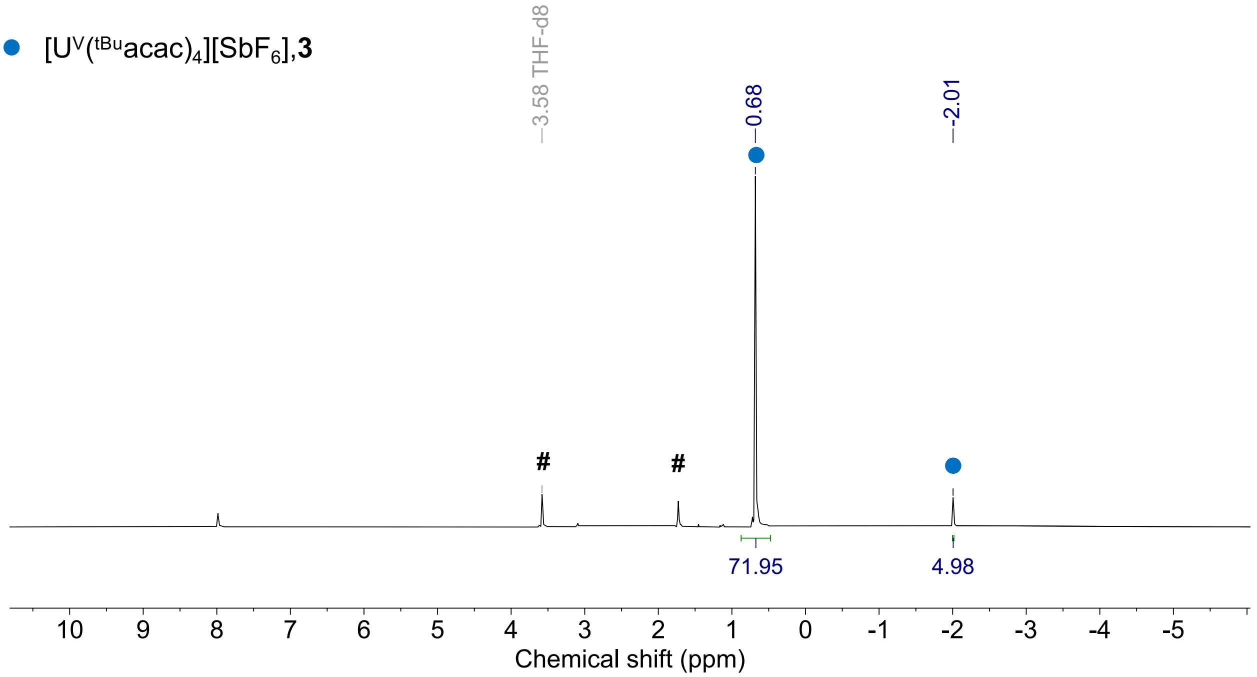

Figure S2: ^{1}H NMR spectrum of [U^{V}(^{tBu}acac)$_4$][SbF$_6$] (**3**) in THFd_8 (#).

UV/Vis/NIR Electronic Absorption Spectroscopy

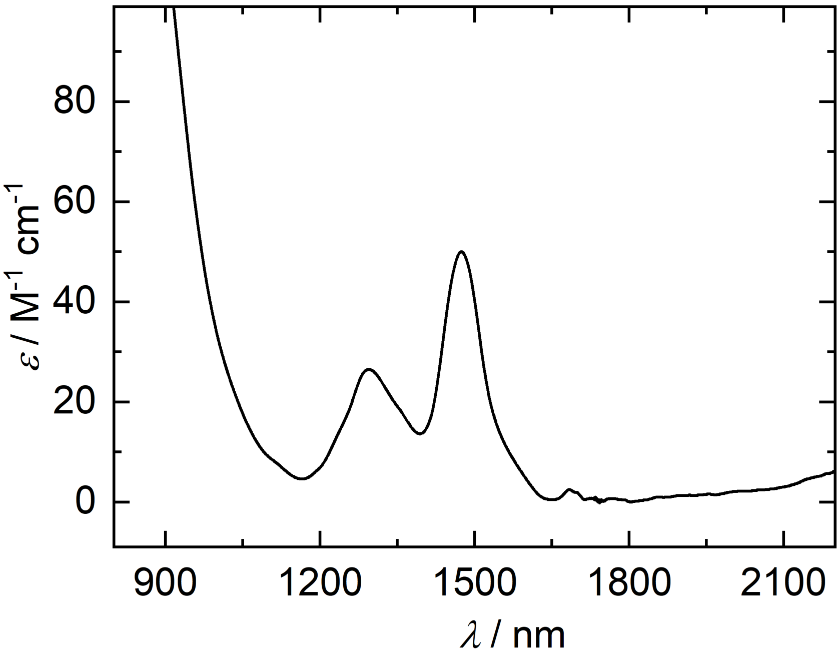

Figure S3: Vis/NIR electronic absorption spectrum of $[U^{V}(^{tBu}acac)_4][SbF_6]$ (**3**) in THF, c = 10 mM.

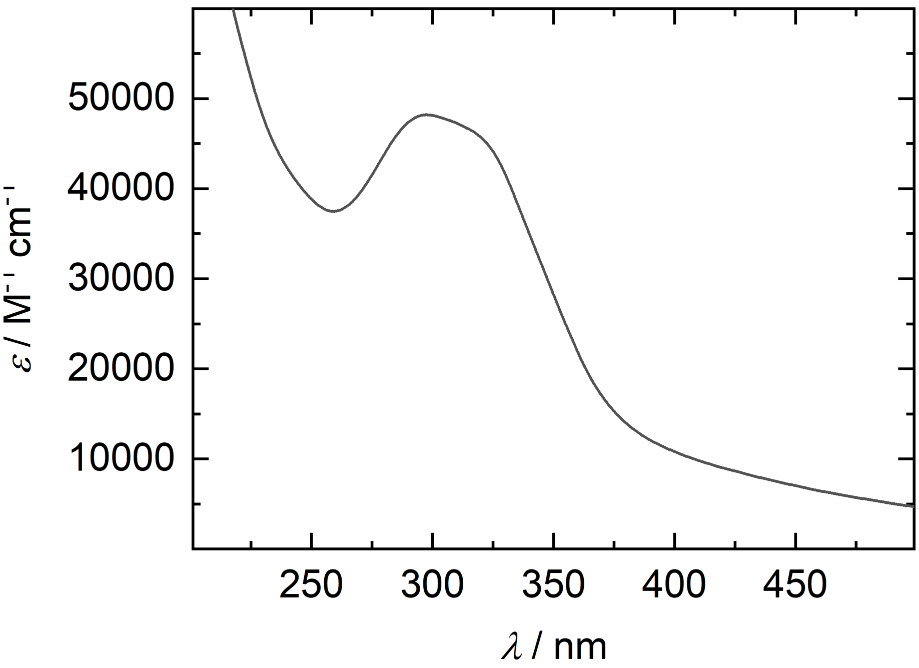

Figure S4: UV/Vis electronic absorption spectrum of $[U^{V}(^{tBu}acac)_4][SbF_6]$ (**3**) in THF, c = 0.1 mM.

Solubility Determination of $[U^{IV}(^{tBu}acac)_4]$ (1) and $[U^{IV}\{N(SiMe_3)_2\}_4]$ (2)

A 50 μM stock solution of $[U^{IV}(^{tBu}acac)_4]$ (**1**) in MeCN was prepared in the glovebox, and a UV-vis spectrum was collected. The solution was then diluted in 10 μL increments, and UV-vis spectra were recorded after each dilution until a final concentration of 10 μL was reached (Figure S5). Absorbances of the five solutions were measured at λ = 298 nm and used to prepare a calibration curve of absorbance as a function of concentration (Figure S6).

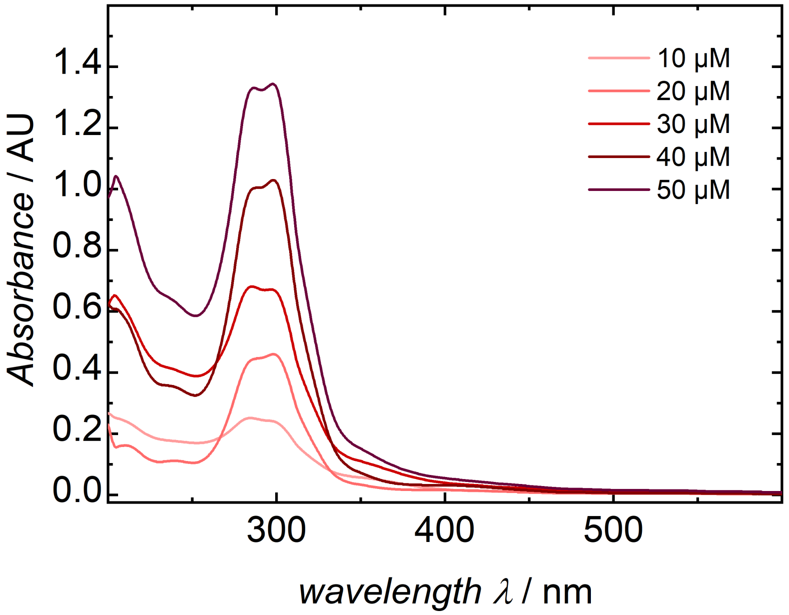

Figure S5: UV-vis spectra of $[U^{IV}(^{tBu}acac)_4]$ (**1**) in MeCN at concentrations in the range 10-50 μM.

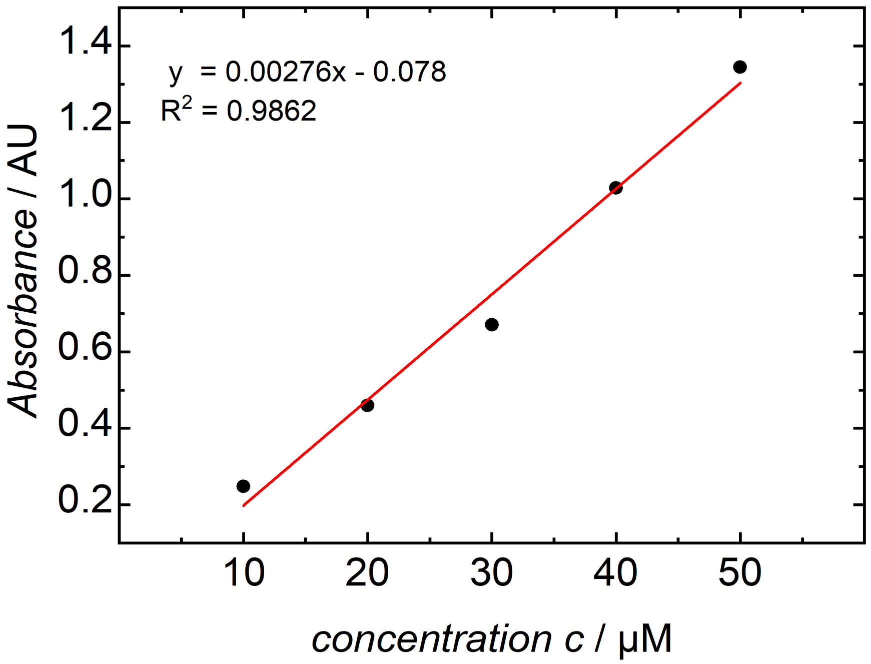

Figure S6: Calibration curve of absorbance as a function of concentration of $[U^{IV}(^{tBu}acac)_4]$ (**1**) in MeCN.

An initial solution of $[U^{IV}(^{tBu}acac)_4]$ (**1**) in MeCN was concentrated until precipitation of **1**, indicating that the solution was saturated. Next, an aliquot of this solution was removed and diluted with MeCN until the absorbance of the resulting solution was lower than 1.0 (final dilution factor was 1:24000).

Five independent samples were taken, and their UV-vis spectra were collected (Figure S7). The concentration of each solution was calculated independently based on the calibration curve previously described.

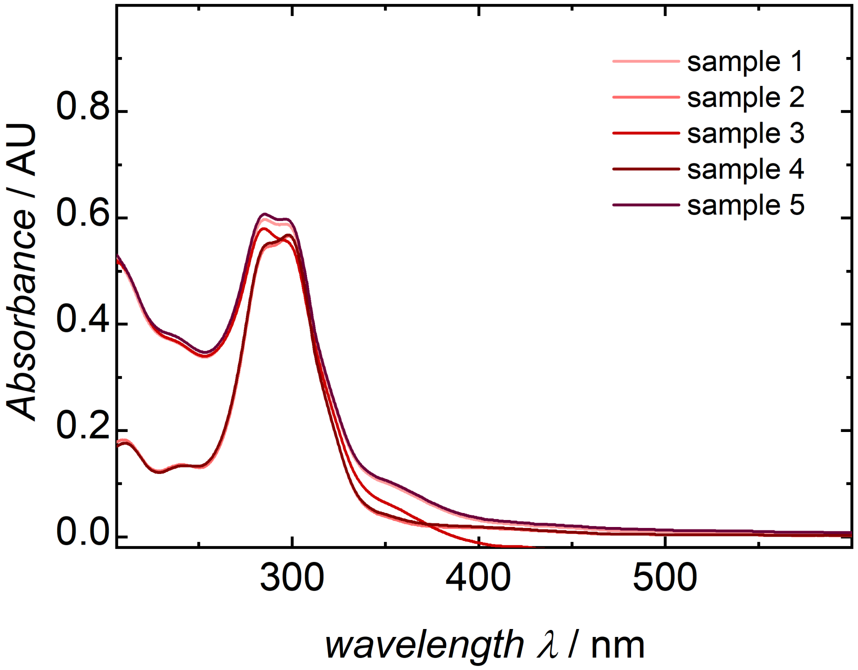

Figure S7: UV-vis spectra of five independent samples of $[U^{IV}(^{tBu}acac)_4]$ (**1**), prepared by dilution (1:24,000) of a saturated solution in MeCN.

Sample	Absorbance	Concentration of diluted sample (mM)	Concentration of saturated sample (mM)
1	0.587	0.0241	578
2	0.566	0.0233	559
3	0.555	0.0229	550
4	0.568	0.0234	561
5	0.597	0.0244	586
average	0.575	0.0236	**567**

Average concentration of saturated solution of 1: 0.567 M

A 150 μM stock solution of $[U^{IV}(N(SiMe_3)_2)_4]$ (**2**) in MeCN was prepared in the glovebox. By serial dilution of appropriate volume, standard concentrations of 50, 75, 100, and 125 μM were prepared. From each dilution, the UV-vis spectrum was collected around the wavelength of the maximum absorption of **2** (λ = 326 nm) (Figure S8).

The calibration curve was constructed based on the absorbances at λ = 326 nm of the five dilutions as a function of concentration (Figure S9).

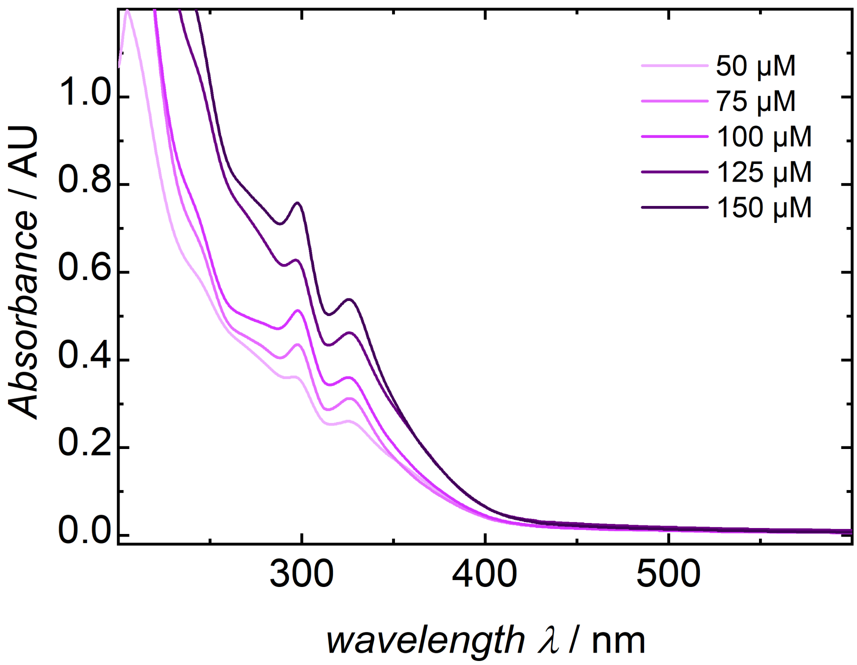

Figure S8: UV-vis spectra of $[U^{IV}(N(SiMe_3)_2)_4]$ (**2**) in MeCN at concentrations in the range 50-150 μM.

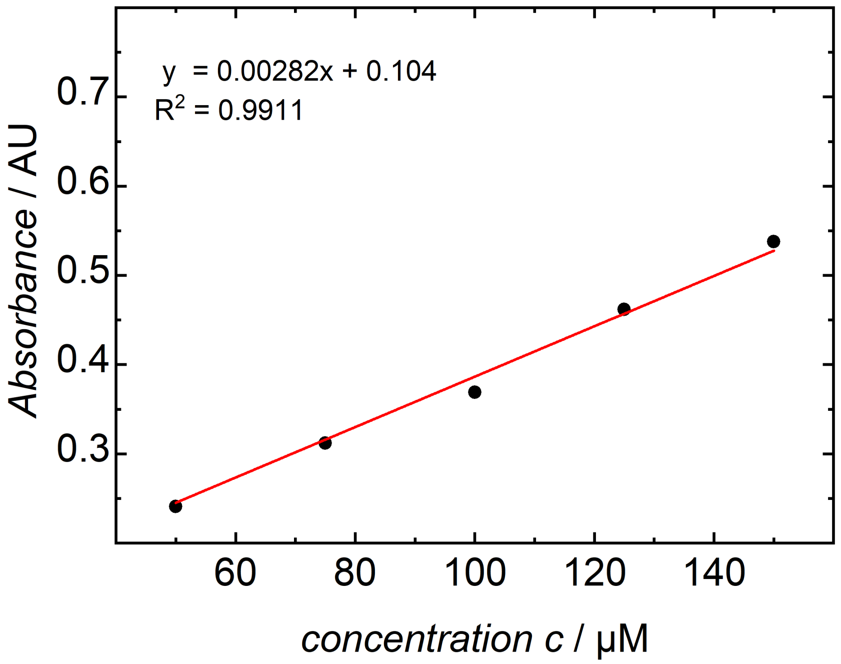

Figure S9: Calibration curve of absorption as a function of concentration of $[U^{IV}(N(SiMe_3)_2)_4]$ (**2**) measured in MeCN.

An initial solution of $[U^{IV}(N(SiMe_3)_2)_4]$ (**2**) in MeCN was concentrated until precipitation of **1**, indicating that the solution was saturated. Next, an aliquot of this solution was removed and diluted with MeCN until the absorbance of the resulting solution was lower than 1.0 (final dilution factor was 1:600).

Five independent samples were taken and their UV-vis spectra were collected (Figure S10). The concentration of each solution was calculated independently based on the calibration curve previously described.

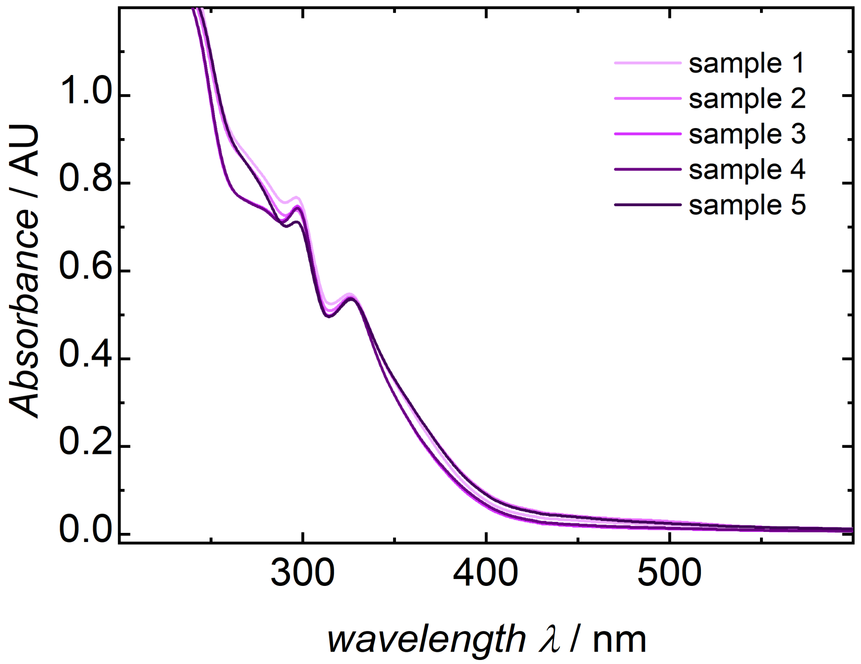

Figure S10: UV-vis spectra of 5 independent samples of $[U^{IV}(N(SiMe_3)_2)_4]$ (**2**) prepared by dilution (1:600) of a saturated solution in MeCN.

Sample	Absorbance	Concentration of diluted sample (mM)	Concentration of saturated sample (mM)
1	0.547	0.157	94.3
2	0.541	0.155	93.0
3	0.538	0.154	92.3
4	0.538	0.154	92.3
5	0.535	0.153	91.7
average	0.540	0.155	**92.8**

Average concentration of a saturated solution of 2: 0.0928 M

VT SQUID Magnetization Data

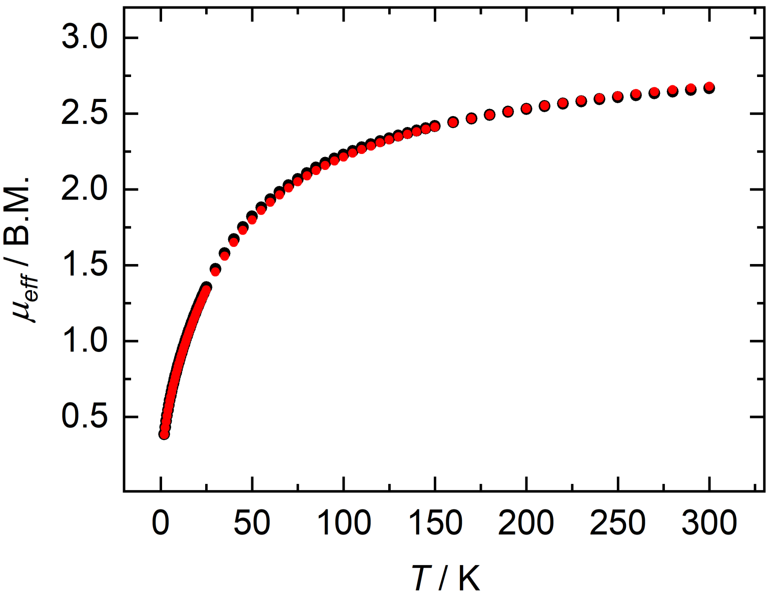

Figure S11: Temperature-dependent SQUID magnetization measurements of $[U^{IV}(^{tBu}acac)_4]$ (**1**); two independently synthesized and studied samples. Data reproduced from ref. [1]

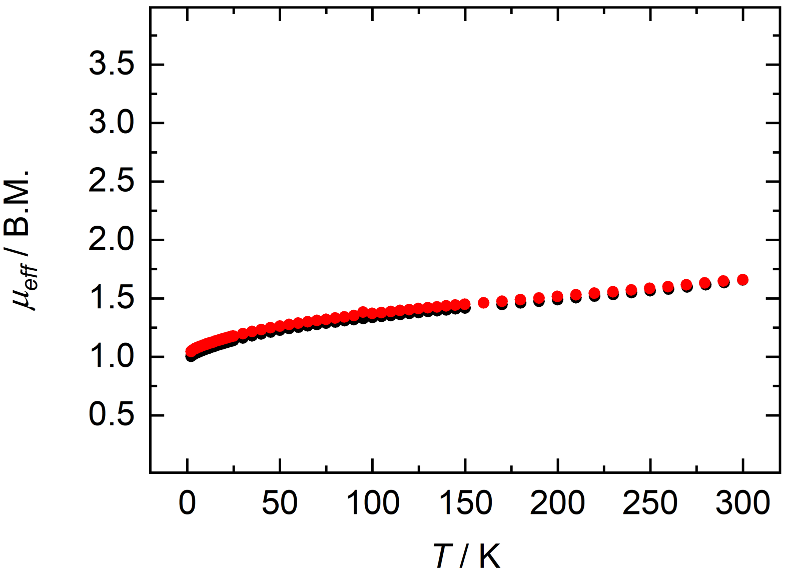

Figure S12: Temperature-dependent SQUID magnetization measurements of $[U^{V}(^{tBu}acac)_4][SbF_6]$ (**3**); two independently synthesized and studied samples.

EPR Spectroscopic Measurements

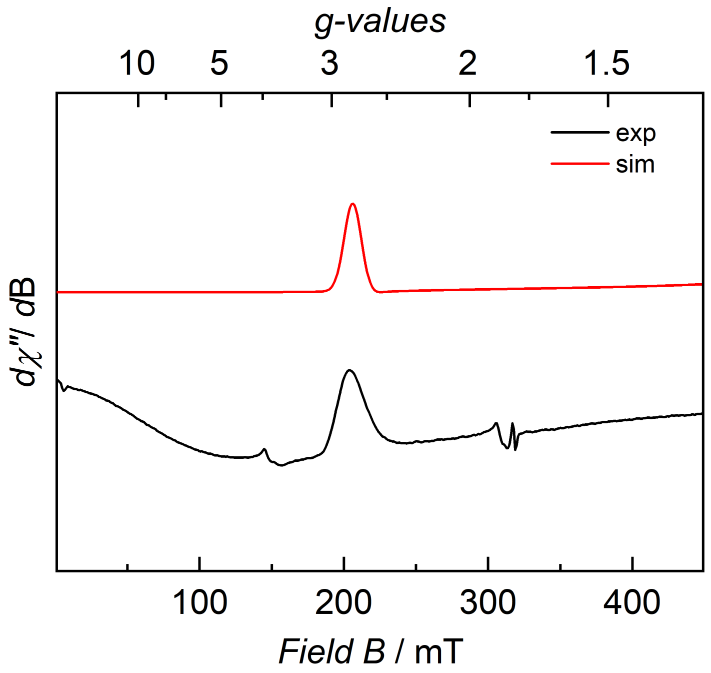

Figure S13: CW X-Band EPR spectrum of $[U^{V}(^{tBu}acac)_4][SbF_6]$ (***3***), recorded at *6* K as a powder (v = 8.96776 GHz, P = 1.0 mW, modulation = 0.1 mT). The spectrum was simulated with g values of g_1 = *3.10* and line widths of W_1 = *12* mT. The g_2 and g_3 values lie outside the experimentally accessible range and were set to arbitrary values for simulation.

Electrochemical Details

General procedure for bulk electrolysis studies.

Charge-discharge tests were performed with an experimental design similar to the one reported by Thompson *et al.* [10] The experiments were performed inside of a glovebox under an inert atmosphere of purified N_2 at 25 °C and in an H-cell composed of two compartments with 5 mL volume each. Each compartment contained graphite electrodes (SynLectro™ from Merck, with 1 cm^2 macroscopic area), with an interelectrode distance of ca. 6 cm, and a Neosepta ACS® anion-exchange membrane (ASTOM, Japan) was used to separate each compartment. [11] The membrane was activated by soaking in a 1.0 M MeCN solution of [TBA][PF_6] for 48 h prior to use. During cycling, contents in both reservoirs of the H-cell were continuously stirred with Teflon-coated stir bars. Galvanostatic charge-discharge experiments were recorded with an Ivium Vertex potentiostat/ galvanostat using IviumSoft software.

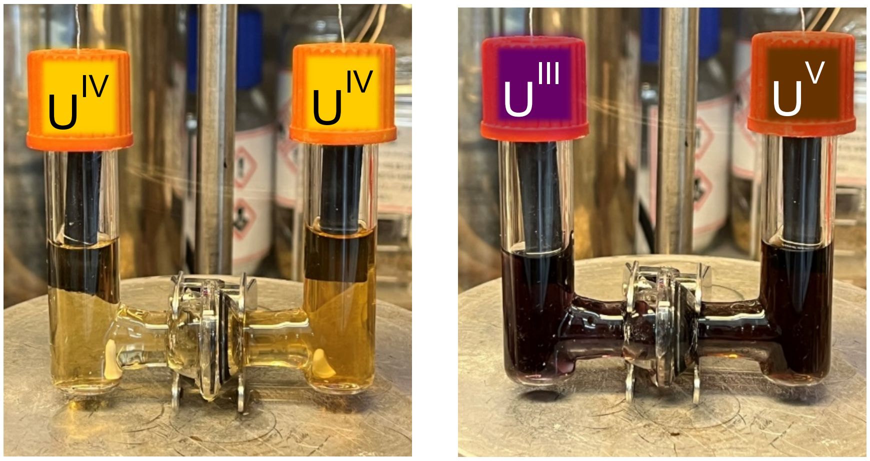

Figure S14: Photographs of the H-cell setup. Initial cell (left), and cell during charging (right).

In the glovebox, $[U^{IV}(^{tBu}acac)_4]$ (**1**) (4.9 mg, 0.005 mmol), $[U^{IV}(N(SiMe_3)_2)_4]$ (**2**) (4.4 mg, 0.005 mmol), and [TBA][PF_6] (400 mg, 1.032 mmol) were weighed in three separate vials. MeCN (10 mL) was used to dissolve [TBA][PF_6], and then half of this solution (5 mL) was used to dissolve $[U^{IV}(^{tBu}acac)_4]$ (**1**) and the other half (5 mL) was used to dissolve $[U^{IV}\{N(SiMe_3)_2\}_4]$ (**2**). Each solution was transferred to a separate compartment of an H-cell, and the cell was galvanostatically charged/discharged at 25 °C in the voltage range of 0.25–2.25V with the following parameters:

Theoretical capacity of cell = 0.000005 mol × 96,485.33 C/mol = 0.482 sA = 0.134 mAh

80% capacity of cell = 0.107 mAh

Stage 1: Galvanostatic charge at 20 μA (until E = 2.25 V)

Stage 2: Galvanostatic discharge at 5 μA (until E = 0.25 V)

Energy density $= 0.0005\,mol \times \frac{1\times 96485.33\frac{c}{mol}\times 2{,}2V}{1L\times 0{,}786\;kg/L} = 135.03\,J/kg = 0.0375\,\frac{Wh}{Kg}$

Max energy density $= 0.0460\,mol \times \frac{1\times 96485.33\frac{c}{mol}\times 2{,}2V}{1L\times 0{,}786\;kg/L} = 12422.8\,J/kg = 3.451\,\frac{Wh}{Kg}$

The maximum energy density was calculated based on the determined concentration of a saturated solution of **2** (0.0928 M) and considering that the two half-cells are prepared separately by dissolution of the complexes.

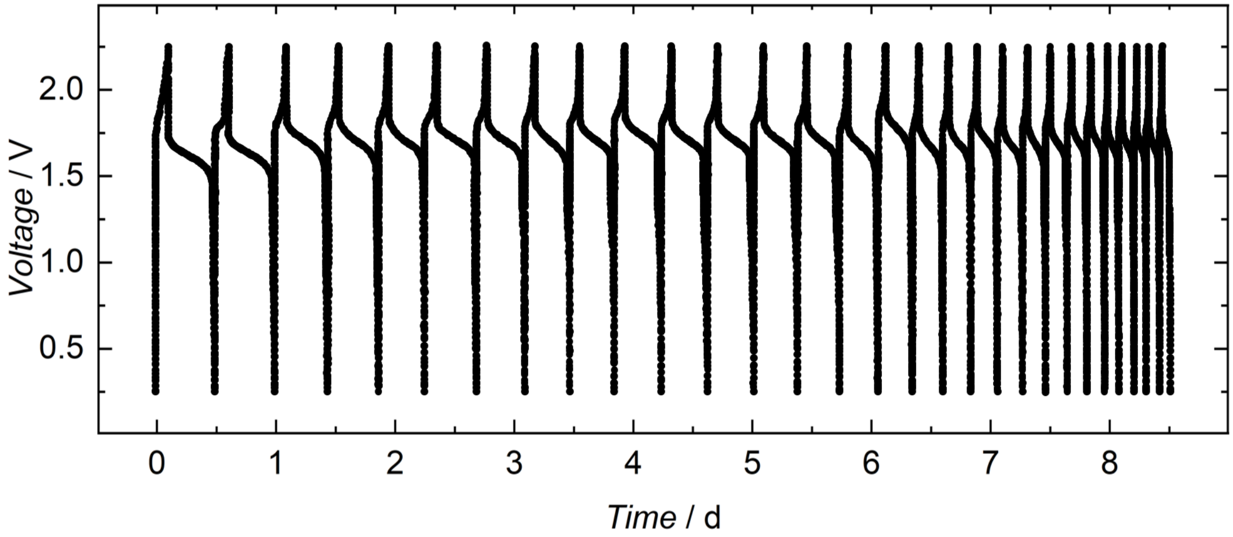

Figure S15: Charge-discharge profile of the 1st–29th cycle of the $[U^{IV}(N(SiMe_3)_2)_4]$(+) | $[U^{IV}(^{tBu}acac)_4]$(−) (**1**||**2**) cell. Galvanostatic cycling of the cell was performed at 20 μA charge/ 5 μA discharge rate at 25 °C under an atmosphere of purified N_2. Conditions: anolyte, 0.02 mM $[U^{IV}(^{tBu}acac)_4]$ in a 0.1 M MeCN solution (5 mL) of $[TBA][PF_6]$; catholyte, 0.02 mM $[U^{IV}(N(SiMe_3)_2)_4]$ in a 0.1 M MeCN solution (5 mL) of $[TBA][PF_6]$. A NeoseptaACS® (ASTOM, Japan) anion-exchange membrane was used to separate each compartment of the H-cell, and each side was equipped with a carbon paper electrode (2 cm^2 surface area).

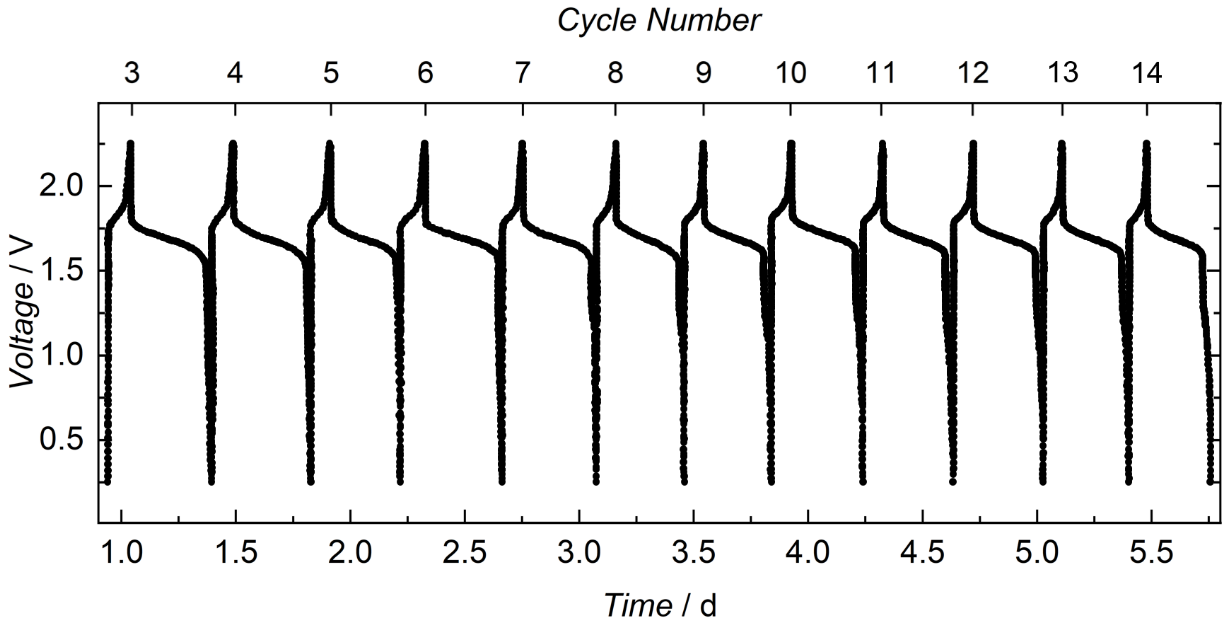

Figure S16: Charge-discharge profile during the 3rd to 14th cycles of the **1**||**2** cell (5 mM in MeCN).

Investigations into the cause of cell capacity decay

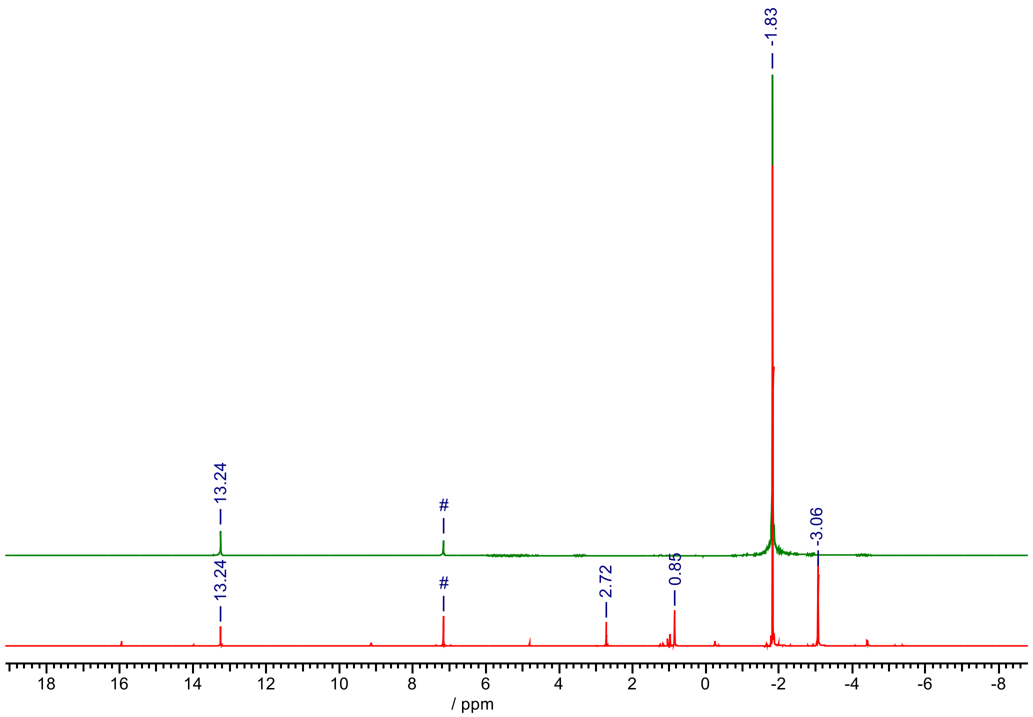

Figure S17: ^{1}H NMR spectra of the anolyte solution in benzene-d_6 (#) before (green) and after (red) 29 charge-discharge cycles.

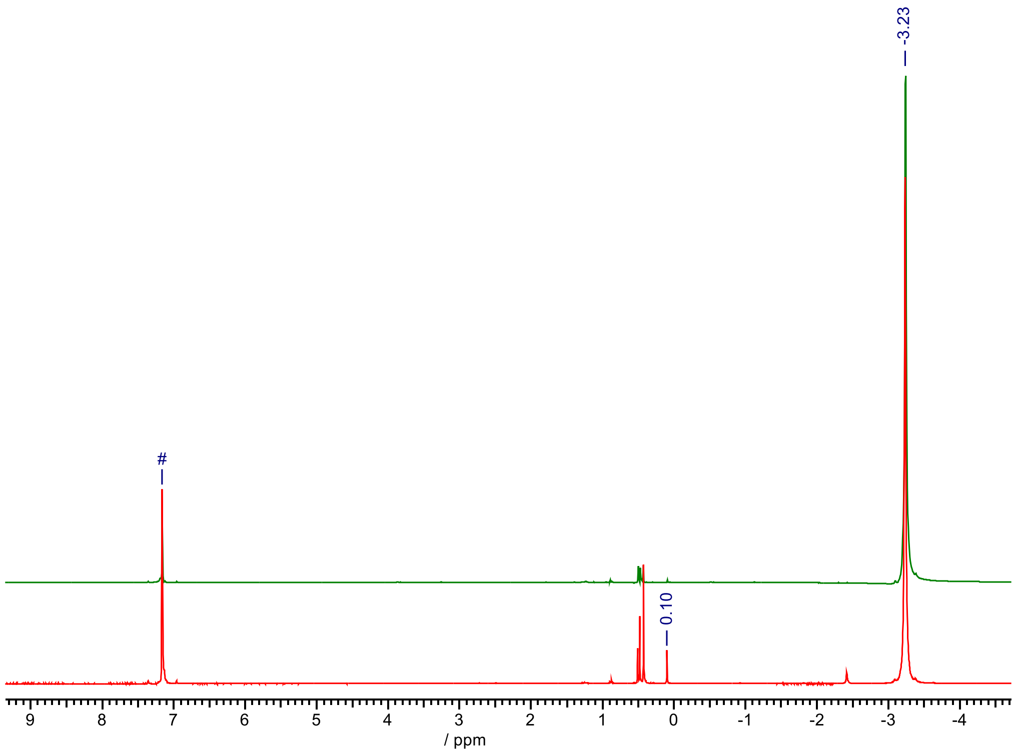

Figure S18: ^{1}H NMR spectra of catholyte solution in benzene-d_6 (#) before (green) and after (red) 30 charge-discharge cycles.

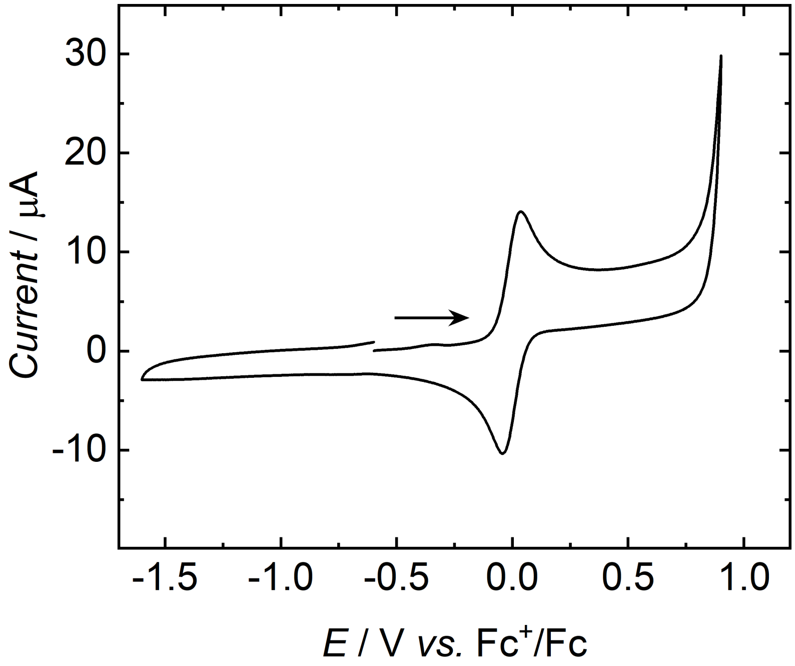

Figure S19: Cyclic voltammogram of Ferrocene with the carbon electrode used in the **1**||**2** cell before 30 charge-discharge cycles, measured in a MeCN solution with ~0.1 M $[N(^nBu)_4][PF_6]$ as an electrolyte; scan rate = 200 mV/s.

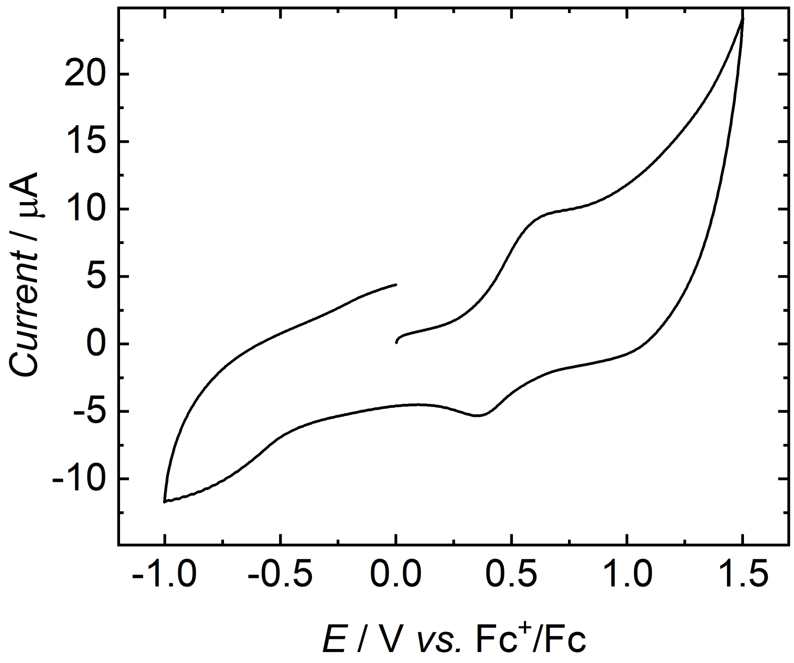

Figure S20: Cyclic voltammogram of ferrocene with the carbon electrode used in the **1**||**2** cell after 30 charge-discharge cycles measured in a MeCN solution with ~0.1 M $[N(^nBu)_4][PF_6]$ as an electrolyte; scan rate = 200 mV/s.

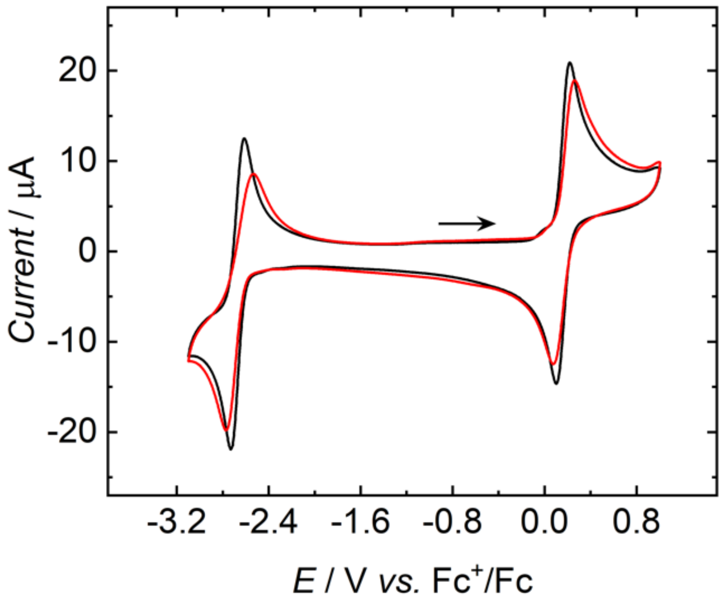

Figure S21: Cyclic voltammograms of [U^{IV}(^{tBu}acac)$_4$] measured in a benzene/MeCN (1:1) solution with ~0.1 M [N(nBu)$_4$][PF_6] as an electrolyte, including the 2nd scan (black) and the 100th scan (red); scan rate = 200 mV/s. Data reproduced from ref. [1]

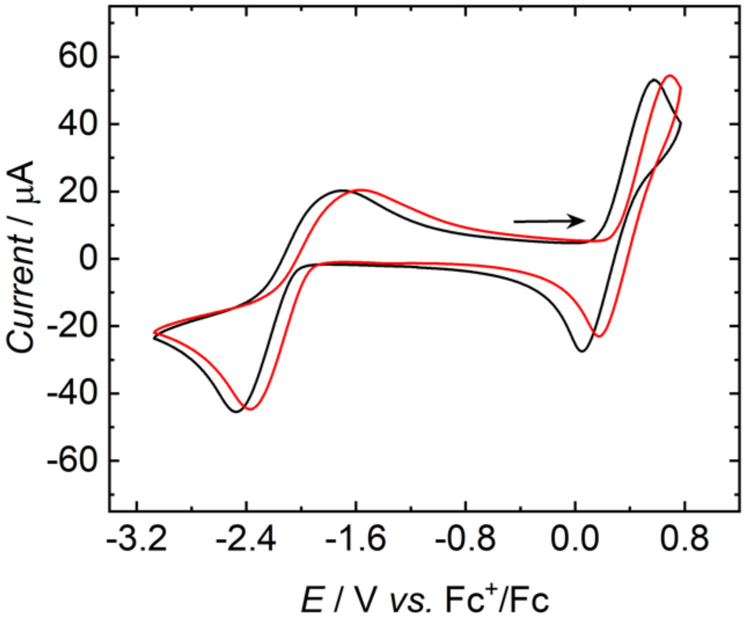

Figure S22: Cyclic voltammograms of $[U^{IV}(HMDS)_4]$ measured in a MeCN solution with ~0.1 M $[N(nBu)_4][PF_6]$ as an electrolyte, including the 2nd scan (black) and the 100th scan (red); scan rate = 200 mV/s

X-Ray Crystal Structure Determination

CCDC-2498207 for **3**, [U^{V}(^{tBu}acac)$_4$][SbF_6] contain the supplementary crystallographic data for this paper. The data can be obtained free of charge from Cambridge Crystallographic Data Centre, 12 Union Road, Cambridge, CB2 1EZ, UK (fax: ++44-1223-336-033; e-mail: deposit@ccdc.cam.ac.uk).

Single crystals of **3** were obtained from diffusion of *n*-pentane into a THF solution at −30 °C and were studied by sc-XRD analysis and are shown in Figure S21. A suitable single crystal of [U^{V}(^{tBu}acac)$_4$][SbF_6] was embedded in protective perfluoropolyalkyether oil on a microscope slide and a single specimen was selected and subsequently transferred to the cold nitrogen gas stream of the diffractometer. Intensity data were collected using MoK_α radiation (λ = 0.71073 Å) on a Bruker Kappa PHOTON2 *IμS* Duo diffractometer equipped with QUAZAR focusing Montel optics. Data were corrected for Lorentz and polarization effects, semi-empirical absorption corrections were performed on the basis of multiple scans using *SADABS*.[12] The structures were solved by direct methods (SHELX XT 2014/5)[13] and refined by full-matrix least-squares procedures on F^2 using SHELXL 2019/3.[14] All non-hydrogen atoms were refined with anisotropic displacement parameters. All hydrogen atoms were placed in positions of optimized geometry, their isotropic displacement parameters were tied to those of the corresponding carrier atoms by a factor of either 1.2 or 1.5. Olex2[15] was used to prepare material for publication. Crystallographic data, data collection, and structure refinement details are given in Table S1.

The investigated crystal turned out to be an inversion twin. The refinement as inversion twin resulted in twin individual mass fractions of 0.821(3) and 0.179(3). One of the *t*-butyl groups in one of the ^{tBu}acac ligands was disordered. Two alternative orientations were refined and resulted in site occupancies of 75.7(8) and 24.3(8) % for the atoms C19 – C22 and C19A – C22A, respectively. The SbF_6 anion was disordered. Two alternative orientations were refined and resulted in site occupancies of 67.6(6) and 32.4(6) % for the atoms F11 – F14 and F11A – F14A, respectively. Similarity restraints were applied in the refinement of the anisotropic displacement parameters of the disordered atoms of the *t*-butyl group and of the atoms F11 and F11A.

The molecular structure of the monocation **3** in crystals of [U^{V}(^{tBu}acac)$_4$][SbF_6] features four ^{tBu}acac ligands bound to the uranium center, confining the uranium ion in an square antiprismatic coordination geometry (Figure S23). The molecular structure further reveals that both the mean distances of the U–O (2.26(7) Å) compare well with the previously reported analogue [U^{V}(^{tBu}acac^{Me})$_4$][SbF_6] (2.240 Å)[1] consistent with the oxidation to U(V). The O–U–O bite angle of the ^{tBu}acac ligand in **3** amounts to 70.7°, which is similar to the one observed in **1** (69.67°) (Table S1).

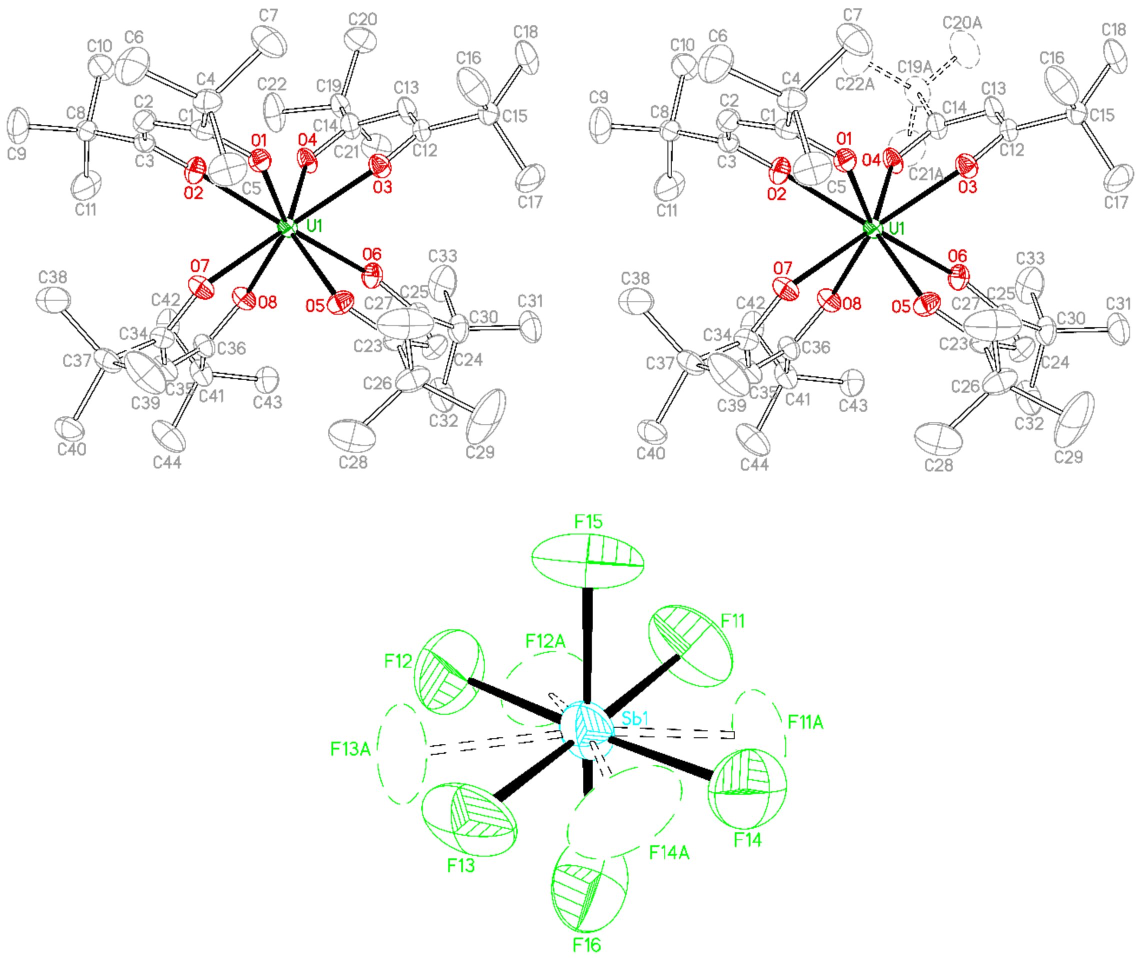

Figure S23: Solid-state molecular structure with the applied numbering scheme of **3** in crystals of $[U^{V}(^{tBu}acac)_4][SbF_6]$ (top left: major component of the $[U^{V}(^{tBu}acac)_4]$ cation, top left: minor component of the $[U^{V}(^{tBu}acac)_4]$ cation, bottom: the SbF_6 anion with the minor component of the disordered atoms drawn with dashes . Thermal ellipsoids are at the 50% probability level, hydrogen atoms are omitted for clarity).

Table S1: Selected Interatomic Distances (Å) and Angles (deg) of $[U^{V}(^{tBu}acac)_4][SbF_6]$ (**3**).

**Parameter**	**3**
U1–O1	2.228(2)
U1–O2	2.255(2)
U1–O3	2.245(2)
U1–O4	2.259(2)
U1–O5	2.156(3)
U1–O6	2.401(2)
U1–O7	2.338(2)
U1–O8	2.191(2)
<O1–U1–O2	71.01(8)
<O3–U1–O4	70.62(8)
<O5–U1–O6	70.52(9)
<O7–U1–O8	70.54(8)

Table S2: Crystallographic data, data collection, and refinement details for **3**.

	**3**<br>CCDC 2498207
Empirical Formula	$C_{44}H_{76}F_6O_8SbU$
Mol. weight [g/mol]	1206.82
Crystal shape,color	block, brown
Crystal size [mm^3]	0.40×0.27×0.20
Temperature [K]	100
Crystal system	Monoclinic
Space group	*Cc*
a [Å]	24.5310(12)
b [Å]	10.5501(5)
c [Å]	19.8962(10)
α [°]	90
β [°]	97.910(2)
γ [°]	90
V [A^3]	5100.2(4)
Z	4
ρ_{calc} [g/cm^3]	1.572
μ [mm^{-1}]	3.768
F(000)	2404.0
T_{min}; T_{max}	0.557; 0.746
2Θ interval [°]	3.3 ≤ 2 Θ ≤ 63.1
Coll. Refl.	75282
Indep. Refl.; R_{int}	16664, 0.0301
Obs. refl. I≥2σ(I)	16189
No. ref. param.	643
wR_2 (all data)	0.0417
R_1 (I > 2σ(I))	0.0183
GooF on F^2	1.034
$\Delta\rho_{max/min}$ [e Å^{-3}]	1.341; –1.093

References

(1) P. Waldschmidt, J. Riedhammer, D. R. Hartline, F. W. Heinemann, K. Meyer, *Inorganic Chemistry* **2023**, *62*, 2013-2023.

(2) A. J. Lewis, U. J. Williams, P. J. Carroll, E. J. Schelter, *Inorganic Chemistry* **2013**, *52*, 7326-7328.

(3) G. A. Bain, J. F. Berry. *J. Chem. Educ.* **2008**, *85* (4), 532–536.

(4) E.Bill, SQUID Program JulX2. 2019.

(5) D. F. Evans. *J. Chem. Soc.* **1959**.

(6) A. Marcó, R. Compañó, R. Rubio, I.Casals. *Mikrochim. Acta* **2003**, *142* (1–2), 13–19.

(7) Bill, E. EPR Program Eview. 2019.

(8) Bill, E. EPR Program Esim. 2019.

(9) B. J.Gaffney, H. J. Silverstone. *Simulation of the EMR Spectra of High-Spin Iron in Proteins*; Springer, Boston, MA, **1993**; pp 1–57..

(10) Q. Liu, A. E. Sleightholme, A. A. Shinkle, Y. Li, L. T. Thompson, *Electrochem. Commun.* **2009**, 11, 2312–2315.

(11) http://www.astom-corp.jp.

(12) L. Krause, R. Herbst-Irmer, G. M. Sheldrick, D. Stalke, Dietmar, *Journal of Applied Crystallography* **2015**, 48, 3-10.

(13) G. M. Sheldrick, *Acta Crystallographica Section A* **2008**, 64, 112-122.

(14) G. M. Sheldrick, *Acta Crystallographica Section C* **2015**, 71, 3-8.

(15) O. V. Dolomanov, L. J. Bourhis, R. J. Gildea, J. A. K. Howard, H. Puschmann, *Journal of Applied Crystallography* **2009**, 42, 339-341.
